# A small-angle X-ray scattering study of red blood cells in continuous flow

**DOI:** 10.1107/S1600577523002011

**Published:** 2023-04-07

**Authors:** Jan-Philipp Burchert, Rita Graceffa, Oliva Saldanha, Manfred Burghammer, Sarah Köster

**Affiliations:** a Institute for X-ray Physics, University of Göttingen, Friedrich-Hund-Platz 1, 37077 Göttingen, Germany; b Cluster of Excellence ‘Multiscale Bioimaging: from Molecular Machines to Networks of Excitable Cells (MBExC)’, University of Göttingen, Germany; c ESRF – The European Synchrotron, 71 Avenue des Martyrs, CS40220, 38043 Grenoble Cedex 9, France; ESRF and Université Grenoble Alpes, France

**Keywords:** red blood cells, SAXS, microfluidics, cells in flow

## Abstract

Owing to their large penetration depth and high resolution, X-rays are ideally suited to study structures and structural changes within intact biological cells. For this reason, X-ray-based techniques have been used to investigate adhesive cells on solid supports. However, these techniques cannot easily be transferred to the investigation of suspended cells in flow. Here, an X-ray compatible microfluidic device that serves as a sample delivery system and measurement environment for such studies is presented. As a proof of concept, the microfluidic device is applied to investigate chemically fixed bovine red blood cells by small-angle X-ray scattering (SAXS). A very good agreement is found between in-flow and static SAXS data. Moreover, the data are fitted with a hard-sphere model and screened Coulomb interactions to obtain the radius of the protein hemoglobin within the cells. Thus, the utility of this device for studying suspended cells with SAXS in continuous flow is demonstrated.

## Abstract 2

Microfluidics enables us to measure whole mammalian cells in continuous flow by small-angle X-ray scattering.

## 1. Introduction

In recent years, X-rays have been employed to study internal structures in biological cells. X-ray scattering, in particular, offers the possibility to obtain structural data from subcellular components, such as proteins or lipids (Hémonnot & Köster, 2017 ▸). The small wavelength, thus high intrinsic resolution, and high penetration power, make them ideally suited to study whole, intact cells without the need for, for example, slicing the sample. Adherent cells are mostly studied while attached to solid supports (Miao *et al.*, 2003 ▸; Dierolf *et al.*, 2010 ▸; Giewekemeyer *et al.*, 2011 ▸; Weinhausen *et al.*, 2012 ▸, 2014 ▸; Nam *et al.*, 2013 ▸; Rodriguez *et al.*, 2015 ▸; Hémonnot *et al.*, 2016 ▸; Bernhardt *et al.*, 2016 ▸; Nicolas *et al.*, 2017 ▸; Deng *et al.*, 2017 ▸; Hémonnot & Köster, 2017 ▸; Cassini *et al.*, 2020 ▸; Wittmeier *et al.*, 2021 ▸, 2022 ▸). Suspension cells may be kept in solution and, for example, filled into glass capillaries or cuvettes (Liu *et al.*, 2015 ▸). Here, we suggest an alternative approach: we present a microfluidic device, in which cells can be investigated by small-angle X-ray scattering (SAXS). During the investigation, the cells flow through the X-ray beam. We demonstrate the utility of the device by applying it to a study of red blood cells (RBCs) that are responsible for the transport of oxygen from the lungs to the tissue and carbon dioxide in the opposite direction. RBCs possess a biconcave disc-like shape and contain large quantities of the protein hemoglobin but no cell organelles. This densely packed hemoglobin solution is enveloped by a cell membrane that is supported by a spectrin–actin network (Alberts *et al.*, 2008 ▸). RBCs of different species differ in size but the internal hemoglobin concentration is highly similar between, for example, ovine, bovine and human cells (Jikuya *et al.*, 1998 ▸; Gregory, 2000 ▸).

Hemoglobin possesses several conformational states between which it can switch, for example, by binding of ligands (Deatherage & Moffat, 1979 ▸; Janin & Wodak, 1993 ▸; Fronticelli *et al.*, 1995 ▸; Safo & Abraham, 2001 ▸; Lukin *et al.*, 2003 ▸; Rusciano, 2010 ▸; Nelson & Goodsell, 2014 ▸). Among these states, the oxy-/‘relaxed’ and the deoxy-/‘tensed’ states are the most important ones for respiratory oxygen and carbon dioxide transport (Surgenor, 1975 ▸; Begemann & Rastetter, 1993 ▸; Nelson & Goodsell, 2014 ▸). Such conformational changes of proteins occur on the nanometer scale and thus SAXS or small-angle neutron scattering (SANS) are ideal techniques to probe them.

The small-angle scattering (SAS) signal of human RBCs and hemoglobin solutions has successfully been measured in bulk experiments. It features a characteristic ‘interaction peak’ or ‘interference peak’, which is visible in concentrated solutions and during measurements with whole cells but not for diluted hemoglobin solutions (Krueger & Nossal, 1988 ▸; Krueger *et al.*, 1990 ▸; Svergun *et al.*, 2008 ▸; Stadler *et al.*, 2010 ▸; Liu *et al.*, 2015 ▸; Shou *et al.*, 2020 ▸). The shape of the peak depends on both the pH value and the hemoglobin concentration (Krueger *et al.*, 1990 ▸; Stadler *et al.*, 2010 ▸). At pH 7.4 (pD 7.4 for heavy water) it can be found at concentrations above 200 g L^−1^ (Krueger *et al.*, 1990 ▸). However, the intensity of the peak, which is usually found at a scattering vector of approximately *q*_*r*_ = 1 nm^−1^, and its exact position depend on the concentration of hemoglobin. Higher hemoglobin concentrations shift the peak to larger *q*_*r*_-values and reduce its intensity, whereas lower salt concentrations shift the peak to smaller *q*_*r*_-values and increase its intensity (Krueger *et al.*, 1990 ▸; Liu *et al.*, 2015 ▸). This behavior is also seen for whole cells (Krueger & Nossal, 1988 ▸; Liu *et al.*, 2015 ▸; Shou *et al.*, 2020 ▸). The only previous SAS measurements of RBCs in flow were performed employing a Couette shear cell with SANS. The authors observed anisotropic scattering at shear rates above 1000 s^−1^ which is suggested to be caused by aggregation of hemoglobin in the RBCs (Garvey *et al.*, 2004 ▸).

Here, we perform a proof-of-concept SAXS study on chemically fixed bovine RBCs using a composite PMDS-glass microfluidic device to keep the cells in suspension and in continuous flow during the measurement. Our results are very much in line with static capillary measurements that we perform as a benchmark and we fit the data to obtain quantitative values for characteristic parameters describing the sample system. Our method opens up the possibility to study virtually all cell types in suspension and in flow and can in principle also make use of the shear flow in the microfluidic field, diffusive mixing-in of reagents, and time-resolved data collection.

## 2. Material and methods

### 2.1. PDMS-capillary device

**Figure 1:**
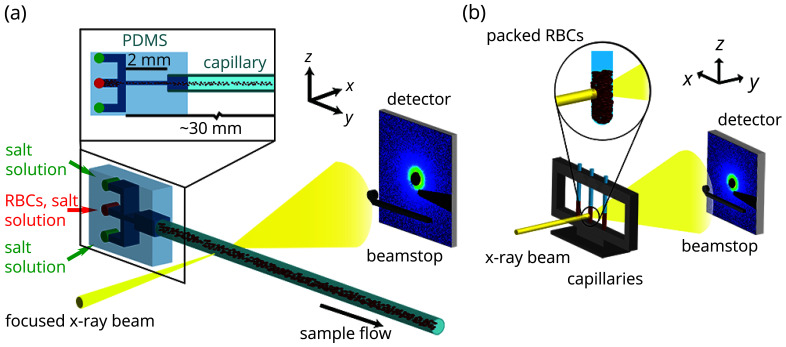
(*a*) Schematic of the PDMS-capillary device and sketch of the setup at ID13, ESRF. Red blood cells (RBCs) in NaCl solution (*c*_NaCl_ = 9 g L^−1^; central inlet) are flow focused by two streams of the same salt solution without cells (side inlets). The focused X-ray beam probes the RBC stream in the quartz glass capillary (inner diameter 180 µm) and the signal is recorded by a 2D pixel detector. (*b*) Schematic of the experimental in-house setup for static measurements. The X-rays probe densely packed RBCs in quartz glass capillaries. The scattering signal is recorded by a 2D pixel detector.

The polydimethylsiloxane (PDMS)-capillary devices (Zheng *et al.*, 2004 ▸) are fabricated by standard soft lithography techniques (Whitesides & Stroock, 2001 ▸) as described in detail elsewhere (Saldanha *et al.*, 2017 ▸). Briefly, a layer of SU-8 3050 (MicroChem, Newton, USA) with a height of 200 µm is spin coated onto a silicon wafer and structured by photolithography to obtain the channel structure presented in Fig. 1 ▸(*a*) with three inlets (*h* × *w* = 200 µm × 100 µm) and one outlet (*h* × *w* = 200 µm × 50 µm, widening to 200 µm × 200 µm). This master structure is used to mold the channel structure into PDMS (Silgard 184, Dow Corning, Midland, MI, USA) and the channels are sealed by a flat PDMS piece. A quartz glass capillary (outer diameter 200 µm, wall thickness 10 µm; Hilgenberg, Malsfeld, Germany) is inserted into the outlet channel and sealed with uncured PDMS. Finally, the whole PDMS-capillary device is cured by baking at 65°C for one hour.

### 2.2. Cell samples

Glutaraldehyde-fixed bovine RBCs (Bioleague GmbH & Co. KG, Poggensee, Germany) are employed for both static and in-flow measurements. The volume fraction of the cells in solution is approximately 10%. The cells are washed three times with 9 g L^−1^ NaCl (Carl Roth, Karlsruhe, Germany) in ultrapure water. This salt solution is also used for both the background measurements of the static experiments and the lateral inlets of the in-flow experiments.

### 2.3. X-ray setup and data analysis for static measurements

Static SAXS experiments are conducted with an in-house setup (Xeuss 2.0, Xenocs, Sassenage, France), see Fig. 1 ▸(*b*). A Cu *K*_α_ source (*E* = 8 keV; Genix 3D, Xenocs, Sassenage, France) is operated at 50 kV and 600 µA. Multilayer optics and scatterless slits reduce the beam to a size of approximately 0.5 mm × 0.5 mm, resulting in an integrated beam intensity of *I*_0_ ≃ 10^7^ counts s^−1^. The scattering signal is recorded at a sample-to-detector distance of 1227 mm by a Pilatus3 R 1M detector (Dectris Baden, Switzerland; 981 × 1043 pixels, pixel size 172 µm × 172 µm). A beamstop with a diameter of 3 mm protects the detector from the incident primary beam. For each quartz glass capillary (Hilgenberg, Malsfeld, Germany) with an outer diameter of 1.5 mm and a wall thickness of 10 µm the following measurement protocol is pursued: transmission measurements (exposures of 0.1 s without beamstop) of (i) the empty and (ii) the water-filled capillary to determine its inner diameter; (iii) the transmission and scattering of the capillary filled with salt solution is measured for 4 h split into 10 min acquisitions; (iv) densely packed RBCs in salt solution are measured using the same exposure scheme. Packing is obtained by centrifuging the cell solution in sealed capillaries at 1200×*g* for 5 min (Eppendorf 5810R, Eppendorf AG, Hamburg, Germany). The measurement is repeated for six capillaries.

We process the scattering data with self-written Matlab (Matlab 9.3, The MathWorks, Natick, MA, USA) scripts, based on the PSI cSAXS Matlab package (https://www.psi.ch/en/sls/csaxs/software). The recorded scattering images are added, masked and azimuthally averaged to obtain radial intensity profiles of the salt solution and the cell suspension for every capillary. The intensity profiles are corrected for their total exposure time and transmission before the radial intensity profile of the salt solution is subtracted from one of the cells. The resulting background-subtracted intensity profiles are divided by the inner diameter of the respective capillary. The intensity profiles are further normalized by the error-weighted average of their intensities in a *q*_*r*_-range of 0.95 ≤ *q*_*r*_ ≤ 1.05 nm^−1^. Finally, we determine an average intensity profile by averaging all the normalized intensity profiles in a point-wise manner.

The model we fit to the radial intensity profiles is based on the product of a prefactor 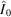 , a form factor *F*(*q*_*r*_) and a structure factor *S*(*q*_*r*_, *r*_HS_, *c*, κ, *Z*) that depends on the molar hemoglobin concentration *c* within the cells, the hard-sphere radius *r*_HS_ of hemoglobin, the inverse of the Debye screening length κ and the number of charges per hemoglobin *Z*,

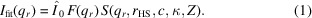

The volume fraction of hemoglobin within the cells is then given as: *n* = 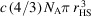 , with the Avogadro number *N*_A_. The form factor is calculated from the crystal structure of bovine deoxy-hemoglobin (PDB entry: 1hda) (Perutz *et al.*, 1993 ▸; Berman *et al.*, 2000 ▸) using the software *FoXS* (https://modbase.compbio.ucsf.edu/foxs/) (Schneidman-Duhovny *et al.*, 2013 ▸, 2016 ▸). It is normalized by its maximum intensity value at *q*_*r*_ = 0 nm^−1^ and linearly interpolated to the *q*_*r*_-values of our experiment. The structure factor models hard-sphere interactions and a screened Coulomb potential of spherical and charged particles in dielectric solutions in the mean spherical approximation. This structure factor is applicable to the concentrated hemoglobin solution within the cell membrane of the RBCs and is directly calculated for the *q*_*r*_-values of our experiment (Hayter & Penfold, 1981 ▸; Pedersen, 1997 ▸). The product *I*_fit_(*q*_*r*_) from prefactor, form factor and structure factor is fitted to the measured radial intensity profile in a *q*_*r*_-range of 0.8 ≤ *q*_*r*_ ≤ 4 nm^−1^ by optimizing the parameters 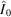 , *r*_HS_, *c*, κ and *Z*. Here, the Levenberg–Marquardt nonlinear least-squares algorithm as implemented in the Matlab function *nlinfit* is applied. All errors of the fitted parameters reflect the 1σ confidence interval.

### 2.4. Synchrotron X-ray setup, in-flow experiment and data analysis

The bovine RBCs within the PDMS-capillary device are measured at the EHII hutch of the ID13 beamline of ESRF (The European Synchrotron, Grenoble, France). Photons with an energy of *E* = 12.6 keV are focused to a beam size of 8 µm × 9 µm to obtain a primary beam intensity of up to *I*_0_ ≃ 10^12^ counts s^−1^. The scattering signal is recorded with an EigerX 4M detector (Dectris, Baden, Switzerland) at a sample-to-detector distance of 769 mm. The experimental setup is sketched in Fig. 1 ▸(*a*).

First, salt solution is filled into the capillary by both lateral inlets at a flow rate of *Q*_l_ = 100 µL h^−1^ for each inlet and the scattering signal of the solution is measured. Second, cell solution is added via the central inlet at a flow rate of *Q*_c_ = 75 µL h^−1^ and the scattering signal of the cells is determined. Scattering data of both the salt solution and the RBCs are acquired at different downstream positions of the capillary with mesh scans (three columns with a distance of 0.01 mm and at least three rows with variable distances). The total exposure time per mesh point of 10.1 s split into 0.1 s intervals. Line scans perpendicular to the capillary (61 steps with a step size of 5 µm, 0.1 s exposure time per position) are performed between the mesh scans to account for movements of the PDMS-capillary device during the experiment.

The scattering images of the salt solution are added, masked, azimuthally averaged and corrected for their total exposure time to obtain a single radial intensity profile for every downstream position. Similarly, radial intensity profiles of RBCs in salt solution are calculated for every mesh point. Moreover, up to three profiles that belong to one row of a mesh scan are averaged. We scale the intensities of the salt solution to those of the RBCs in salt solution before background subtraction such that they coincide for *q*_*r*_ > 7.6 nm^−1^. The resulting background-subtracted radial intensity profiles of the RBCs are normalized, averaged and fitted as described for the static measurements. For the whole analysis, scattering images that contain strong streaks or data pairs, where the salt solution signal exceeds the cell signal, are excluded as this is likely caused by strong movement of the capillary between the measurements.

### 2.5. Finite-element method simulations

We conduct finite-element methods (FEM) simulations using *COMSOL Multiphysics* (version 5.5; COMSOL, Stockholm, Sweden) to investigate the flow conditions inside the PDMS-capillary device. To reduce the computational effort the device geometry is simplified as follows. (i) The two symmetry planes of the geometry are exploited such that only one quarter of the device is simulated. (ii) The constriction is reduced from a length of 2 mm to a length of 100 µm, as the diffusion of the cells is negligible. (iii) The flow in the capillary is simulated only for a length of about 1.6 mm. The simplified geometry is created with *AutoCAD* (Autodesk, Munich, Germany) and imported into *COMSOL*. We simulate the RBC concentration within the PDMS-capillary device with the ‘transport of diluted species’ module (*D*_RBC_ = 1.35 × 10^−13^ m^2^ s^−1^ and *c* = 2.86 × 10^−12^ mol L^−1^ 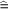 0.1 L L^−1^) and couple it to the velocity field that is calculated for single phase laminar flow. Due to the applied symmetries the flow rates are adjusted to *Q*_l_/2 = 50 µL h^−1^ (water, lateral inlets) and *Q*_c_/4 = 18.75 µL h^−1^ (RBCs in water, central inlet). In contrast to the experiments, we do not add salt in the simulations as the salt concentration is equal everywhere in the device. We apply a tetrahedral mesh with an edge length between 0.2 µm and 1.5 µm for the junction, the constriction and the first 100 µm of the capillary. The rest of the device is simulated with a coarser mesh (edge length between 13 µm and 63 µm). Velocity and RBC concentration profiles are extracted from the stationary result of the simulation. As we employ chemically fixed cells at a low concentration of 10% vol/vol, non-Newtonian effects, for example, caused by deformation of the RBCs (Shevkoplyas *et al.*, 2006 ▸; Guo *et al.*, 2014 ▸; Abay *et al.*, 2019 ▸), can be neglected in our simulation. Moreover, our simulation does not include any cell–cell interactions or the influence that the excluded volume of the cells has on the flow field.

## 3. Results and discussion

### 3.1. Static capillary measurements

**Figure 2:**
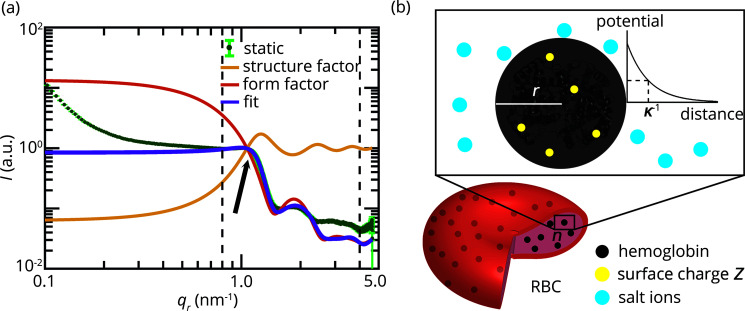
(*a*) Background-corrected, normalized, averaged radial intensity profile from the hemoglobin within bovine RBCs, static experiments (*N* = 6). Model fit (purple) to the average profile from the static measurements (green). The model is fitted to the data between *q*_*r*_ = 0.8 nm^−1^ and *q*_*r*_ = 4.0 nm^−1^, indicated by the vertical, dashed lines. The model is based on a prefactor, a form factor of bovine deoxy-hemoglobin (red, PDB:1hda) and a structure factor (yellow). The black arrow indicates a steep decay of the signal which is typical of hemoglobin and in some literature work shows up as a peak. (*b*) Illustration of the different components and parameters of the fit model. The form factor is given by the structure of deoxy-hemoglobin. To obtain a structure factor, the hemoglobin molecules are modeled as hard spheres (black) with radius *r* at a volume fraction *n* inside the RBCs. Moreover, the hemoglobin molecules carry charges *Z* (yellow circles) which are screened (κ) by salt ions (blue circles) in the cytoplasm.

The high concentration of hemoglobin in RBCs gives rise to a distinct small-angle scattering signal (Krueger & Nossal, 1988 ▸; Krueger *et al.*, 1990 ▸; Garvey *et al.*, 2004 ▸; Svergun *et al.*, 2008 ▸; Liu *et al.*, 2015 ▸). As a benchmark for our in-flow measurements, we conduct static measurements of densely packed RBCs in quartz glass capillaries. We split up the total exposure time of 4 h into 10 min intervals and observe no differences between the earlier and later exposures. Thus, we conclude that no radiation-induced sample damage occurs beyond the first 10 min. We average six scattering profiles stemming from individual measurements, as shown in Fig. 2 ▸(*a*) (green data points).

Several authors report very similarly shaped intensity profiles from SANS and SAXS measurements for experiments with RBCs or concentrated hemoglobin solutions (Guinier & Fournet, 1955 ▸; Krueger & Nossal, 1988 ▸; Krueger *et al.*, 1990 ▸; Garvey *et al.*, 2004 ▸; Stadler *et al.*, 2010 ▸; Liu *et al.*, 2015 ▸). The intensity profile shows a decay of the intensity in the *q*_*r*_-range 0.1 < *q*_*r*_ < 0.3 nm^−1^. The decay is followed by a plateau-like region and another steep decay at approximately 1.0 nm^−1^, as indicated by the black arrow in Fig. 2 ▸(*a*). The position of this decay in our measurements agrees with the value from other SAXS measurements, where it sometimes appears as a peak (Krueger & Nossal, 1988 ▸; Liu *et al.*, 2015 ▸), but is found at a slightly larger *q*_*r*_-value compared with findings from SANS measurements (Krueger & Nossal, 1988 ▸). Finally, oscillations with multiple local minima and maxima are observed at approximately *q*_*r*_ ≥ 1.5 nm^−1^. The same oscillations are also found in diluted hemoglobin solutions (Svergun *et al.*, 2008 ▸; Liu *et al.*, 2015 ▸). Consequently, these oscillations are mostly a result of the form factor of hemoglobin, whereas the *q*_*r*_-value of the transition from the plateau to the steep decay is a result from the interference of the decay of the form factor and the increase of the structure factor of hemoglobin, which is highly concentrated within RBCs (Krueger & Nossal, 1988 ▸; Krueger *et al.*, 1990 ▸; Garvey *et al.*, 2004 ▸; Svergun *et al.*, 2008 ▸; Shou *et al.*, 2020 ▸).

In contrast to our experiments, where we use fixed bovine RBCs, the aforementioned studies use unfixed human RBCs. However, we do not expect large differences in the intensity profiles stemming from the RBCs of both species. Firstly, human and bovine RBCs have the same mean corpuscular hemoglobin concentration (Jikuya *et al.*, 1998 ▸). Therefore, the volume fractions of the RBCs are the same. Secondly, the hemoglobin molecules have very similar diffusion coefficients (Jones *et al.*, 1978 ▸; Bouwer *et al.*, 1997 ▸), indicating a similar size. Finally, the differences in structure, amino acid composition and hydrophobic properties are small (Perutz *et al.*, 1993 ▸; Fronticelli *et al.*, 1995 ▸). It is therefore reasonable to assume that both types of RBCs scatter in a very similar manner.

To quantify and compare our intensity profiles with the literature, we fit our data with a structure factor based on a screened Coulomb potential (Krueger & Nossal, 1988 ▸; Krueger *et al.*, 1990 ▸; Liu *et al.*, 2015 ▸) and a form factor calculated from the known hemoglobin structure (Perutz *et al.*, 1993 ▸; Berman *et al.*, 2000 ▸), as described in Section 2.3. Our fit parameters are presented in Table 1 ▸ and illustrated in Fig. 2 ▸(*b*). The hard-sphere radius *r*_HS_ and the charge *Z* are properties of the hemoglobin molecules. The charge that the molecules carry depends, for example, on the pH value of the surrounding medium. The composition of the cytoplasm of the RBCs is characterized by the volume fraction of hemoglobin molecules *n* and by the concentration of salt ions. The screening of the charges of the hemoglobin is characterized by the Debye screening coefficient κ in our model.

Although our fit captures the *q*_*r*_-values of the minima and maxima in the radial intensity profile well, it deviates in terms of intensity magnitudes, especially for intensities at *q*_*r*_ > 1.5 nm^−1^. We took care to employ the form factor model for deoxy-hemoglobin, because glutaraldehyde fixation and a low pH due to dissolved CO_2_ switch hemoglobin to its deoxy state (Johnson, 1987 ▸; Abay *et al.*, 2019 ▸). The deviations may stem from scattering contributions of the cell membrane and cortex that are not accounted for by our model, from sample preparation (Minetti *et al.*, 2013 ▸) and from polydispersity of the system.

Comparing our measurements with the literature, we find a good agreement, especially given the variation already reported in previous work (Krueger & Nossal, 1988 ▸; Krueger *et al.*, 1990 ▸; Stadler *et al.*, 2010 ▸; Liu *et al.*, 2015 ▸; Shou *et al.*, 2020 ▸). The deviations for *n*, where we obtain a larger value, and *r*_HS_, which is smaller for our system, go hand in hand and may be related to a slight shrinkage of the cells. In our scattering data, this may be observed as the shift of the steep decay to a slightly larger *q*_*r*_-value, since a larger volume fraction reduces the distances between the hemoglobin molecules. Based on the good agreement between our data from static capillary measurements and the existing literature values, we can readily use the static data as a benchmark for the in-flow data.

### 3.2. Characterization of the flow

**Figure 3:**
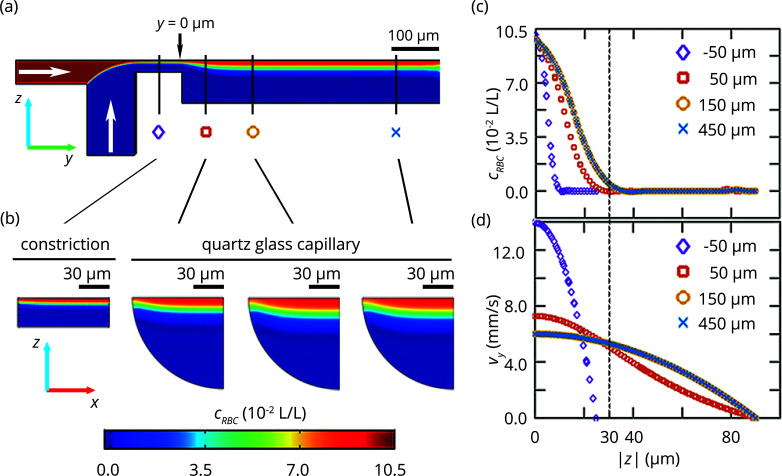
FEM simulations of the flow conditions and RBC volume fraction (vol/vol) inside the PDMS-capillary device. (*a*) Simulated RBC concentration [see legend in (*b*)] in the central *y*–*z*-plane of the device. RBCs are injected into the central inlet while water is injected into the side inlets (white arrows). Here we replace the salt solution, as used in the experiment, by pure water. The fluids flow through a constriction and enter the quartz glass capillary at *y* = 0 µm. (*b*) Cross sections (*x*–*z*-plane) at different downstream positions along the main flow direction. The first cross section shows the red blood cell concentration in the constriction; the following three cross sections show the concentration in the capillary. (*c*) Simulated RBC concentration profiles and (*d*) velocity profiles along the *z*-axis at the downstream positions that are marked in (*a*) by the respective symbols.

Microfluidic setups offer excellent control of the experimental conditions. To make full use of this control, however, a thorough characterization of the flow conditions is a necessary prerequisite. Specifically, to interpret the in-flow data, a precise knowledge of the velocity field and the spatial distribution of the RBCs in the PDMS-capillary device is important. We determine these parameters numerically from FEM simulations. For numerical reasons we simulate a reduced geometry of the PDMS-capillary device and make use of its symmetry. The RBC solution [central inlet from the left hand side in Fig. 3 ▸(*a*)] is flow focused by the lateral inlets (inlet from the bottom; note that we show only the bottom half of the simulation in the figure) at the junction and enters a constriction which in principle would support diffusive mixing of chemical agents. However, we do not use this option for our current proof-of-concept experiments. Finally, the fluid expands into the quartz glass capillary, where the X-ray measurements are conducted to reduce the background noise to a minimum. We model the RBC concentration within the device as a diluted species, which is certainly an approximation that does not cover complex dynamics and cell shapes that are related to the non-Newtonian behavior of RBCs in flow, such as tank threading or cross streamline migration (Fåhraeus & Lindqvist, 1931 ▸; Wells & Merrill, 1962 ▸; Guyton, 1969 ▸; Abkarian *et al.*, 2008 ▸; Coupier *et al.*, 2008 ▸; Munn & Dupin, 2008 ▸; Wan *et al.*, 2011 ▸). However, it is justified as we use fixed RBCs, which are less deformable (Shevkoplyas *et al.*, 2006 ▸; Abkarian *et al.*, 2008 ▸; Guo *et al.*, 2014 ▸; Abay *et al.*, 2019 ▸), and thus do not show these dynamics. Moreover, we use a small volume fraction of just 10%, which is in the application limits of the Comsol interface ‘transport of diluted species’ (COMSOL, 2019 ▸). We approximate an upper boundary for the diffusion coefficient by assuming spherical particles with a radius that corresponds to half of the smallest extension of an RBC *d*_RBC_, taking into account the known volume *V*_RBC_ = 58 µm^3^ and length of the major axis 2*r*_RBC_ = 5.9 µm of a bovine RBC (Gregory, 2000 ▸),

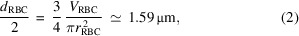




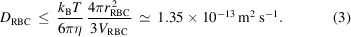

Here, 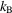 is the Boltzmann constant, *T* = 293 K is the measurement temperature and η = 1 mPa s is the viscosity of water.

As expected for large objects like cells, the diffusion coefficient of the RBCs is very small and diffusion of RBCs from the central stream to lateral positions can be neglected on the time scale of this experiment. This is clearly seen from the FEM simulation shown in Fig. 3 ▸(*a*), where the the RBC stream (a red coloring denotes a high cell concentration) remains in the center of the channel. Therefore, the RBC stream width is governed only by the flow rates at the inlets and the device geometry. The channel cross sections presented in Fig. 3 ▸(*b*) and the line scans in Fig. 3 ▸(*c*) show that a steady-state stream with a constant width is reached at about 150 µm downstream from the constriction. To estimate the width of the RBC stream in the capillary and to account for numerical errors that lead to small deviations from a concentration 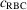 of zero outside the RBC stream, we use 5% of the initial RBC concentration as a threshold. Thus, we obtain a width of approximately 60 µm and can expect an X-ray signal of the cells within approximately 30 µm from the center of the capillary.

The flow velocity of the RBCs in the capillary and through the X-ray beam determines the exposure time for the individual cells. As expected from Hagen–Poiseuille’s law the velocity within the capillary with circular cross section develops a parabolic profile with a maximum velocity of *v*_max_ = 6.0 mm s^−1^, see Fig. 3 ▸(*d*). The corresponding Reynolds number is 1.1, as calculated from the density of water, ρ = 1000 kg m^−3^, the maximum velocity, the viscosity of water, η = 1 mPa s, and the inner diameter of the capillary, *L* = 180 µm.

Using the RBC stream width and the velocity profile [Figs. 3 ▸(*c*), 3(*d*)] from the simulation, the number of cells that contribute to our scattering data and the exposure time per cell in the X-ray experiment can be estimated. The average velocity of the RBCs is obtained by averaging the velocity profile within the width of the RBC stream: 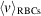 = 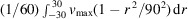 ≃ 5.8 mm s^−1^. With the average velocity and the width of the X-ray beam in the direction of the capillary *b*_h_ = 9 µm, it follows that a single cell spends approximately 1.6 ± 0.1 ms within the beam. With the inner diameter of the capillary *L* = 180 µm, the beam size *b*_v_ × *b*_h_ = 8 µm × 9 µm, the cell density *n* = 0.1 L L^−1^ and the volume of an RBC *V*_RBC_ = 58 µm^3^ (Gregory, 2000 ▸), we estimate the number of cells in the beam at a time, 

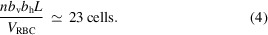

Consequently, multiplying the number of cells in the beam by the ratio of the total exposure time and the exposure time per cell yields the number of cells that contribute on average to each background-subtracted scattering image, *i.e.* ∼4.2 × 10^5^ cells. For the static experiments and assuming dense packing of the RBCs, because RBCs are very flexible, a similar estimate yields 

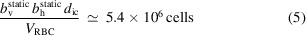

that contribute to the signal. Here, the beam dimensions from the static measurements 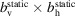 = 0.5 mm × 0.5 mm and the experimentally obtained average of the inner diameter of the capillaries *d*_ic_ = 1.26 mm as well as the volume of an RBC *V*_RBC_ = 58 µm^3^ are used. Clearly, the dynamic measurements considerably reduce the exposure time per individual cell. The microfluidic experiments additionally offer the possibility to fine-tune parameters, such as the number of cells contributing to one scattering image and the total exposure time per cell by the cell concentration, the flow profile and the recording time.

### 3.3. In-flow measurements

During the static SAXS measurements on RBCs as described in Section 3.1 the RBCs spend the whole exposure time in the X-ray beam and ensemble averaging is performed on a limited number of cells. Although we do not observe any radiation-induced changes in the signal beyond 10 min it is possible that, within these first 10 min, radiation damage does already occur. Thus, we here establish a method which allows for SAXS measurements on suspended cells while they flow through the X-ray beam. Consequently, and as outlined in Section 3.2, each cell spends only approximately 1.6 ms in the beam using the parameters of the current study. Moreover, the microfluidic sample delivery method allows us to fine tune the experimental conditions, such as device geometry, flow rates, buffer types and concentrations. In this context, tuning the exposure time per cell might be especially beneficial for experiments with cells at very brilliant X-ray sources, such as the EBS (Extremely Brilliant Source) upgraded European Synchrotron (ESRF) in Grenoble, France. We can thereby account for the ‘dilemma’ of biological matter between dealing with weak scatters, which require a long exposure time, and sensitivity to radiation damage, which would require a short exposure time.

Experimentally, we obtain 13 background-subtracted intensity profiles (from measurements at 38 different mesh points) after processing the recorded data. All these intensity profiles clearly show the steep decay at the same *q*_*r*_-value. Here, we select profiles acquired within a distance of 30 µm from the center of the capillary. In good agreement with the simulation, measurements beyond that distance do not show the steep decay and are thus not included.

The absolute intensities of these profiles differ in a non-systematic manner, which might be caused by fluctuations in the RBCs stream during the experiment. Strong form factor oscillations (*q*_*r*_ > 1.5 nm^−1^) are found in the red blood cell stream while these oscillations are not visible at the edge of the RBC stream, where fewer RBCs contribute to the signal. To compare the in-flow measurements with our static measurements, we normalize and average the 13 intensity profiles, as described in Section 2.

**Figure 4:**
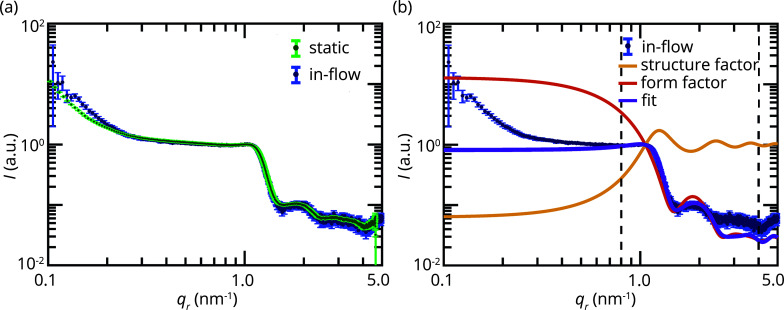
(*a*, *b*) Background-corrected, normalized, averaged radial intensity profiles from hemoglobin within bovine RBCs. (*a*) Comparison of the average profiles from static (green, *N* = 6) and in-flow (blue, *N* = 13) measurements. *N* refers to the number of samples, each of which contains a large number of cells. (*b*) Model fit (purple) to the average profile from the in-flow measurements (blue). The data are fitted between *q*_*r*_ = 0.8 nm^−1^ and *q*_*r*_ = 4.0 nm^−1^, indicated by the vertical, dashed lines. The model is based on a prefactor, a form factor of bovine deoxy-hemoglobin (red, PDB:1hda) and a structure factor (yellow).

The averaged radial intensity profiles from our in-flow and static measurements agree very well in shape, as can be seen in Fig. 4 ▸(*a*), including the plateau, the steep decay and the form factor oscillations of the characteristic signal of RBCs. Small deviations are observed at the initial decay for small *q*_*r*_-values. At these small values, scattering from the beamstop, effects from background subtraction (since we subtract an average background intensity profile), and small differences in the cell sample may contribute to the scattering signal. The origin of the latter has not entirely been understood yet, but several authors suggest experimental conditions such as sample preparations, protein purity, aging (Liu *et al.*, 2006 ▸; Stradner *et al.*, 2006 ▸; Mandal *et al.*, 2014 ▸) or superstructures (Garvey *et al.*, 2004 ▸; Stadler *et al.*, 2010 ▸) that might contribute to the appearance of the decay. For this reason, intensity values at *q*_*r*_ < 0.8 nm^−1^ are not considered in our fit.

The parameters of our fit to the average in-flow profile agree very well with those parameters that we obtained from static experiments, see Table 1 ▸. Fig. 4 ▸(*b*) shows the measured data once more (blue symbols) and the fit in purple. The structure factor and form factor are presented separately in yellow and red, respectively. To summarize, we obtain the same quantitative results from both static and in-flow measurements of fixed RBCs in flow.

In direct comparison, the error bars of the measured, averaged in-flow data are slightly larger than for the static, averaged data and the same is true for the errors of the fit parameters. In view of the number of cells contributing to one data point, *i.e.* 5.5 × 10^6^ versus 32.4 × 10^6^ for in-flow versus static data, this is very reasonable. Moreover, Fig. 4 ▸(*a*) shows that the error bars for *q*_*r*_ > 1.3 nm^−1^ are particularly large. This is because including those normalized intensity profiles from in-flow profiles that feature the steep decay but do not show the form factor oscillations add noise to the average intensity profile.

## 4. Summary and conclusions

In conclusion, we present an in-flow SAXS measurement on suspended cells and use the highly distinct signal from hemoglobin inside intact RBCs as a benchmark for the microfluidic device which we employ. Comparing the SAXS data obtained from in-flow and static SAXS measurements on the same sample system, we find very good agreement based on the shapes of the intensity profiles. Both profiles agree within the range of their errors for a *q*_*r*_-range of about 0.2 nm^−1^ ≤ *q*_*r*_ ≤ 5.0 nm^−1^, show the steep decay at *q*_*r*_ ≃ 1 nm^−1^, and intensity oscillations at *q*_*r*_ > 1 nm^−1^. For quantification, we fit the intensity profiles with a screened Coulomb potential with hard-sphere interactions and the results from the fits are in line with literature values for RBCs or hemoglobin solutions. Furthermore, we can clearly distinguish the contributions of the form factor and the structure factor to the SAXS signal of dense hemoglobin inside the RBCs. The microfluidic device we present here offers a number of advantages as compared with static experiments in standard glass capillaries: first, the exposure to X-ray photons per individual cell is greatly reduced thus diminishing the risk of radiation damage; second, the number of cells contributing to the ensemble average can be tuned by varying the cell concentration, the flow rates and the exposure time; third, cells can additionally be exposed to shear flow fields during the measurement to study the effect of shape changes (Shou *et al.*, 2020 ▸; Garvey *et al.*, 2004 ▸); fourth, cells can in principle be exposed to different buffers or reagents during the measurement; fifth, and related to that, the device allows for time-resolved studies of cellular processes once unfixed samples are employed.

## Figures and Tables

**Table 1:** Fit results for the average intensity profiles of the static and the in-flow measurements Shown are the hard-sphere radius *r*_HS_, the volume fraction of hemoglobin *n*, the inverse Debye screening length κ, the surface charge *Z* and the prefactor 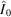 from our fits. The last two columns include corresponding literature values and references.

	Static	In-flow	Literature	
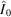 (arbitrary units)	13.4 ± 0.2	13.0 ± 0.3		
*r*_HS_ (nm)	2.57 ± 0.03	2.58 ± 0.05	2.78 ± 0.01	(Krueger & Nossal, 1988 ▸)
			2.75 to 3.04	(Krueger *et al.*, 1990 ▸)
			3.06 ± 0.04	(Svergun *et al.*, 2008 ▸)
			2.97 ± 0.01	(Stadler *et al.*, 2010 ▸)
			2.77	(Liu *et al.*, 2015 ▸)
			2.26	(Shou *et al.*, 2020 ▸)
*n* (L L^−1^)	0.34 ± 0.02	0.34 ± 0.03	0.223 ± 0.002	(Krueger & Nossal, 1988 ▸)
			0.27	(Liu *et al.*, 2015 ▸)
			0.249 ± 0.001	(Shou *et al.*, 2020 ▸)
κ (nm^−1^)	2.82 ± 0.03	2.5 ± 0.8	1.29	(Krueger & Nossal, 1988 ▸)
			1.28	(Liu *et al.*, 2015 ▸)
*Z*	9 ± 14	8 ± 20	12 ± 1	(Krueger & Nossal, 1988 ▸)
			5	(Liu *et al.*, 2015 ▸)
			18 ± 1	(Shou *et al.*, 2020 ▸)
